# Geographical variation in the prevalence of obesity, metabolic syndrome, and diabetes among US adults

**DOI:** 10.1038/s41387-018-0024-2

**Published:** 2018-03-13

**Authors:** Matthew J. Gurka, Stephanie L. Filipp, Mark D. DeBoer

**Affiliations:** 1 Department of Health Outcomes and Policy, College of Medicine, University of Florida, Gainesville, FL 32608 USA; 2 Department of Pediatrics, Division of Pediatric Endocrinology, University of Virginia, PO Box 800386, Charlottesville, VA 22908 USA

## Abstract

Cardiovascular disease (CVD) and type 2 diabetes remain significant public health concerns. Targeting of prevention efforts by geographical location has been suggested by the Institute of Medicine to coincide with the presence of area-based risk. The metabolic syndrome (MetS) is a stronger risk factor than is obesity for the prediction of future CVD and diabetes, yet its prevalence has not previously been described geographically. Our objective is to determine geographical variation in the prevalence of obesity, MetS, and diabetes among US adults. We assessed the prevalence of obesity, MetS, and diabetes by US census division, and the prevalence of obesity, MetS, and diabetes for each sex and racial/ethnic group by US region among 9826 US non-Hispanic white, non-Hispanic black, and Hispanic adults aged 20–65 years participating in the National Health and Nutrition Examination Survey 1999–2014. We also compared a sex- and race/ethnicity-specific MetS severity score by geographical area. The prevalence of obesity, MetS, and diabetes varied by US census division and region, with overall similarity by geographical area in the prevalence of each of these conditions. The prevalence of MetS was particularly high (≥35%) in the West North Central, West South Central, and East South Central and low (30%) in the Pacific, New England, and Mid-Atlantic divisions. Some of the geographical variation appeared due to differences among non-Hispanic white females, who had a high prevalence of MetS (>32%) in the Midwest and South and a low prevalence of MetS (24%) in the West and Northeast. Geographical differences in MetS imply variation in the risk for future CVD and diabetes, with more elevated risk in the center of the United States. As MetS is a stronger risk factor for prediction of CVD and T2DM than is obesity, these differences are potentially important for prompting public health efforts toward surveillance and prevention in high-risk areas.

## Introduction

The epidemic of obesity in the United States and worldwide has raised public health alarm because of associations with multiple comorbidities, including cardiovascular disease (CVD) and type 2 diabetes. This has led to assessments of the geographical distribution of obesity in the United States^1,2^, in part to alert health departments and clinicians in highly affected areas regarding needs for surveillance and preventive services^3^. Nevertheless, not all individuals with obesity have high risk for these chronic diseases^4^. And while some geographical assessments report the geographical distribution of CVD and diabetes themselves; prevalence of these is strongly influenced by access to medical care, which varies widely by region^2^.

One well-described condition potentially in the causative pathway between obesity and CVD and diabetes is the metabolic syndrome (MetS)^5–7^, a cluster of CVD risk factors including central obesity, high blood pressure (BP), high triglycerides, low HDL-cholesterol, and high fasting blood glucose^8^. These abnormalities appear to be produced by underlying processes of systemic inflammation, oxidative stress, and cellular dysfunction^9^. Prior studies demonstrated that individuals with MetS have 2–20-year odds ratios of 1.58 for future CVD^10^ and 5.16 for diabetes^11^. In addition to use of the traditional dichotomous criteria, where MetS is classified if an individual has at least three risk factors (which has been linked to racial/ethnic discrepancies^12–16^), MetS can be assessed using a continuous severity score, which is also linked to risk for future CVD^5,17^ and diabetes^18,19^. Although many international organizations have recognized the potential importance of MetS in risk prediction^7^, geographical variation in MetS has not previously been reported, presumably because it requires a specific set of laboratory and clinical measures to determine. Given the potential to reduce MetS-related risk through primary prevention approaches^20^, knowledge of geographical variation in MetS may be useful to guide prevention measures.

Our goal was to evaluate MetS prevalence by area in the United States on an age-, sex-, and race/ethnicity-specific basis and compare these to the geographical prevalence of (1) obesity and diabetes, (2) MetS severity scores, and (3) the individual MetS components. These data may have implications for emphasis of preventive measures against CVD and diabetes by geographical area.

## Materials/subjects and methods

Data were obtained from the National Health and Nutrition Examination Survey (NHANES) 1999–2014, a complex, multistage probability sample of the US population. These annual cross-sectional surveys are conducted by the National Center for Health Statistics (NCHS) of the Centers for Disease Control; the NCHS ethics review board approved the survey and participants provided informed consent. Participants answered questionnaires and underwent measures of WC, blood pressure (BP), and laboratory measures of fasting triglycerides, HDL-C, and fasting glucose were obtained using standardized protocols and calibrated equipment (http://www.cdc.gov/nchs/nhanes.htm). Analyses were performed at an NCHS Research Data Center due to use of geographic variables. Survey procedures in SAS were used to estimate rates across the nine US census divisions (New England, Mid-Atlantic, East North Central, West North Central, South Atlantic, East South Central, West South Central, Mountain, and Pacific); due to sample size restrictions, rates by sex and race/ethnicity were reported across the four census regions (Northeast, Midwest, South, and West). The groupings of the US census divisions into regions are shown in Supplementary Figure 1. Adults 20–65 years of age were included in the analysis; participants were excluded if reporting they were pregnant or if they were missing any variables related to MetS, diabetes status, BMI, or geographic location (Supplementary Figure 2).

Obesity was defined as BMI ≥30. MetS was defined using the ATP-III criteria^8^. Participants had to meet ≥3 of the following five criteria: elevated waist circumference (≥102 cm for men, ≥88 cm for women), elevated fasting triglycerides (≥150 mg/dl), reduced HDL (<40 mg/dl for men, <50 mg/dl for women), elevated BP (≥130 mmHg systolic or ≥85 mmHg diastolic or use of antihypertensive medication), and elevated fasting glucose (≥100 mg/dl). These criteria did not take diabetes medications or diabetes status (beyond elevated fasting glucose) into account. Diabetes was defined by participant report or fasting glucose ≥126 mg/dl.

MetS severity *z*-scores were calculated using sex- and race-based formulas. As described elsewhere^21–23^, these scores were derived using a confirmatory factor analysis approach for the five traditional MetS components (WC, triglycerides, HDL-cholesterol, systolic BP, and fasting glucose) to determine the weighted contribution of each components to a latent MetS “factor” on a sex- and race/ethnicity-specific basis. Confirmatory factor analysis was performed among adults aged 20–64 years from NHANES 1999–2010 with categorization into six subgroups based on sex and race/ethnicity (non-Hispanic white, non-Hispanic black, and Hispanic). For each of these six population subgroups, loading coefficients for the five MetS components were transformed into a single MetS factor and used to generate equations to calculate a standardized MetS severity score for each subgroup (http://mets.health-outcomes-policy.ufl.edu/calculator/). The resulting MetS severity scores are *z*-scores (normally distributed and ranging from theoretical negative to positive infinity with mean = 0 and SD = 1) of relative MetS severity on a sex- and race/ethnicity-specific basis. These scores correlate strongly with other markers of MetS risk, including hsCRP, uric acid, and the homeostasis model of insulin resistance^22^, with adiponectin^24^ and with long-term risk of CVD^5, 17^ and T2DM^18, 19^. These scores are particularly unique in being specific to sex and to the three major racial/ethnic groups and in being associated with CVD and T2DM independent of the individual MetS risk factors, potentially demonstrating links to risks associated with the processes underlying MetS^5, 19^.

### Statistical analysis

Survey procedures using SAS 9.4 (Cary, NC) were used to account for the complex survey design of NHANES. These procedures were used to estimate rates of obesity, ATP-III MetS, and diabetes, along with mean MetS *z*-scores and BMI, by various demographic subgroups as well as by US census division and region.

## Results

### Participant characteristics by US census division

Table 1 displays participant demographics for adults aged 20–65 years by US census division and region. Notably, in the New England and the West North Central divisions, the mean age tended to be slightly older (44.2 and 44.8 years, respectively—with all others 40.6–41.9 years) and with a greater proportion of non-Hispanic white individuals (92.9 and 93.4%—all others 59.4–84.2%), while the South Atlantic and East South Central had the highest proportion of non-Hispanic black participants (22.8 and 21.4%—all others 1.5–13.4%) and the West South Central and Pacific had the highest proportion of Hispanic participants (28.3 and 28.1%—others 1.7–21.6%).

**Table 1:** Demographic distributions within US census divisions and US census regions among adults aged 20–65 years

		Sex: Male		Age (years)		NHW^a^		NHB^a^		Hispanic	
	*n* ^a^	%^b^	95 CI	Mean	95 CI	%	95 CI	%	95 CI	%	95 CI
US census division											
New England	309	51.6	(45.3, 58.0)	44.2	(42.7, 45.6)	92.9	(88.9, 96.8)	1.5	(0.1, 2.8)	5.7	(2.5, 8.9)
Mid-Atlantic	1265	51.0	(47.8, 54.2)	41.9	(40.5, 43.2)	73.2	(64.4, 82.0)	13.4	(8.2, 18.6)	13.4	(7.6, 19.2)
East North Central	1401	50.5	(48.1, 53.0)	41.5	(40.7, 42.3)	84.2	(79.8, 88.6)	10.6	(7.2, 13.9)	5.3	(3.3, 7.2)
West North Central	542	51.7	(47.7, 55.6)	44.8	(43.8, 45.8)	93.4	(89.3, 97.5)	4.9	(1.5, 8.3)	1.7	(0.5, 2.8)
South Atlantic	2119	49.3	(47.5, 51.0)	41.8	(40.9, 42.7)	65.8	(59.1, 72.5)	22.8	(17.9, 27.6)	11.4	(6.8, 16.0)
East South Central	269	47.0	(43.3, 50.7)	41.9	(39.7, 44.1)	75.6	(65.2, 85.9)	21.4	(12.3, 30.4)	3.1	(0.5, 5.7)
West South Central	1432	50.7	(47.7, 53.7)	40.9	(39.7, 42.2)	59.4	(49.8, 69.0)	12.3	(8.5, 16.1)	28.3	(19.0, 37.6)
Mountain	680	52.7	(48.5, 56.9)	40.6	(38.3, 42.9)	75.8	(66.2, 85.5)	2.6	(0.9, 4.3)	21.6	(13.0, 30.2)
Pacific	1809	50.4	(48.6, 52.2)	41.0	(39.9, 42.0)	66.0	(59.7, 72.4)	5.9	(3.9, 8.0)	28.1	(22.6, 33.5)
US census region^c^											
Northeast	1574	51.2	(48.2, 54.2)	42.5	(41.4, 43.5)	78.2	(71.7, 84.7)	10.4	(6.6, 14.1)	11.4	(7.0, 15.9)
Midwest	1943	50.9	(48.8, 53.0)	42.6	(41.8, 43.3)	87.2	(83.8, 90.6)	8.7	(6.2, 11.3)	4.1	(2.7, 5.5)
South	3820	49.6	(48.1, 51.0)	41.5	(40.7, 42.3)	64.4	(59.3, 69.5)	19.2	(16.0, 22.4)	16.4	(12.2, 20.6)
West	2489	51.2	(49.5, 52.9)	40.8	(39.8, 41.9)	69.3	(64.0, 74.6)	4.8	(3.3, 6.3)	25.9	(21.3, 30.5)

*NHW* non-Hispanic white, *NHB*non-Hispanic black, *CI* confidence interval

^a^ Unweighted (*n*) presented

^b^ Weighted percentages and weighted 95% confidence intervals presented

^c^ Regions are comprised of US census divisions as follows: Northeast: New England and Mid-Atlantic; Midwest: East North Central and West North Central; South: East South Central and West South Central; West: Mountain and Pacific

### Prevalence of obesity, MetS, and diabetes by US census division

**Fig. 1:**
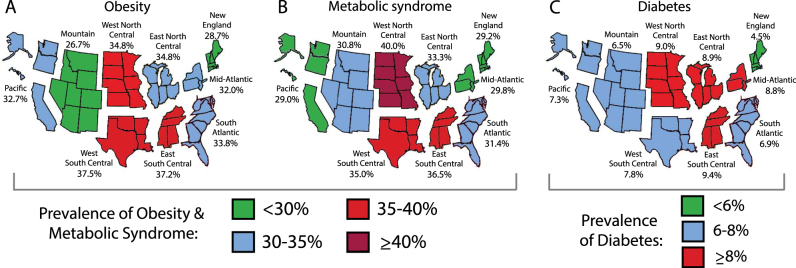
Prevalence of obesity, metabolic syndrome, and diabetes by US census division. Data shown for prevalence of **a** obesity, **b** metabolic syndrome, and **c** diabetes are among US adults aged 20–65 years, from the National Health and Nutrition Examination Survey, 1999–2014

Figure 1 displays prevalence of obesity, MetS, and diabetes for adults overall and by division, and Table 2 provides these data divided into adults aged 20–39 and 40–65 years. Overall, obesity prevalence was lowest in the Mountain and New England divisions (26.7 and 28.7%, respectively) and highest in the West South Central and East South Central (37.5 and 37.2%) (Fig. 1a). Among younger adults, obesity prevalence was low in New England (17.7%), high in the West South Central and East South Central (35.8 and 36.2%), and similar in all other divisions (all 26–29%) (Table 2). Among older adults, obesity was lowest in the Mountain division (27.1%), highest in the divisions that comprise the Midwest and South regions (all ≥38%), and similar in the other divisions (all 35–37%). The difference in prevalence of obesity between younger and older adults was sharp in the divisions in the Northeast and Midwest regions, where older age ranges had >10% higher prevalence than the lower age range.

**Table 2:** Prevalence of obesity, ATP-III MetS, and diabetes by age range and US region

Age group	*N*	Obesity		ATP-III MetS		Diabetes	
		%	95 CI	%	95 CI	%	95 CI
New England							
20–39	116	17.7	(9.1, 26.4)	12.8	(5.3, 20.2)	1.0	(0.0, 2.5)
40–65	193	34.9	(26.8, 43.0)	38.5	(30.0, 47.0)	6.4	(3.6, 9.3)
Mid-Atlantic							
20–39	534	26.1	(21.0, 31.2)	18.8	(14.2, 23.3)	4.4	(3.1, 5.8)
40–65	731	36.6	(31.5, 41.7)	38.4	(32.5, 44.3)	12.2	(8.7, 15.6)
East North Central							
20–39	632	28.8	(24.1, 33.5)	18.8	(15.2, 22.4)	2.6	(1.3, 3.9)
40–65	769	39.6	(35.8, 43.4)	44.9	(40.9, 49.0)	14.0	(11.8, 16.2)
West North Central							
20–39	194	27.2	(17.8, 36.5)	26.8	(18.1, 35.6)	1.5	(0.0, 3.0)
40–65	348	42.0	(35.5, 48.5)	47.0	(38.5, 55.5)	13.0	(9.1, 16.9)
South Atlantic							
20–39	878	29.3	(24.9, 33.7)	17.6	(14.5, 20.8)	2.3	(1.2, 3.4)
40–65	1241	37.4	(33.0, 41.8)	42.2	(38.7, 45.8)	10.4	(8.3, 12.5)
East South Central							
20–39	110	36.2	(22.8, 49.5)	27.3	(19.3, 35.2)	1.2	(0.0, 2.7)
40–65	159	38.0	(24.2, 51.7)	43.9	(32.6, 55.1)	15.9	(10.1, 21.7)
West South Central							
20–39	600	35.8	(28.3, 43.3)	23.5	(17.3, 29.7)	3.1	(1.3, 4.9)
40–65	832	40.6	(35.1, 46.2)	45.7	(39.9, 51.5)	12.1	(9.6, 14.7)
Mountain							
20–39	306	26.2	(19.9, 32.4)	20.3	(16.5, 24.1)	1.7	(0.0, 3.6)
40–65	374	27.1	(22.3, 31.9)	39.9	(33.6, 46.2)	10.7	(4.8, 16.6)
Pacific							
20–39	772	29.5	(25.3, 33.6)	18.7	(15.2, 22.3)	2.6	(1.2, 4.0)
40–65	1037	35.7	(31.8, 39.6)	38.3	(34.7, 41.8)	11.5	(8.7, 14.4)

MetS prevalence overall was lowest in the Pacific and New England (29.0 and 29.2%) and highest in the West North Central (40.0%) (Fig. 1b). Among younger adults, MetS was lowest in New England (12.8%) and highest in the West North Central and the divisions in the South region (all 23–27%) and similar in other divisions (all 18–20%) (Table 2). Among older adults, MetS prevalence was highest in South Atlantic and the divisions in the Midwest and South regions (all 42–47%) and similar in other divisions (all 38–40%).

Diabetes prevalence was lowest in New England (4.5%) and highest in the East South Central and West North Central (9.4 and 9.0%). Among young adults, diabetes prevalence was highest in the Mid-Atlantic and West South Central (4.4 and 3.1%), but overall low in other divisions (all ≤2.6%) (Table 2). Among older adults, diabetes was lowest in New England (6.4%) and high in East South Central and East North Central (15.9 and 14.0%) and similar in other divisions (all 10–13%).

### Sex and racial/ethnic variation by region

We next set out to investigate possible explanations for the geographical variation in MetS, including variation by sex, race/ethnicity, and individual MetS component. Supplementary Table 1 provides sex- and racial/ethnic-specific breakdown by US region. There were notable differences among non-Hispanic white individuals across regions. In the Northeast and West, non-Hispanic white females had favorable prevalences of obesity (25.8 and 30.3%, respectively), MetS (23.3 and 24.9%), and diabetes (4.1 and 4.4%), while in the West, non-Hispanic white males also had low prevalence of obesity (25.7%), MetS (32%), and diabetes (6%). By contrast, in the Midwest non-Hispanic whites (overall, 87% of the Midwest population) had a high prevalence of obesity (males, 34.2%; females, 35.2%), MetS (males, 34.2%; females, 35.2%), and diabetes (males, 10.3%; females, 7%). In the South, there was a high prevalence of MetS among non-Hispanic white males (36.2%) and among Hispanic females (34.4%). Non-Hispanic black males had high prevalence of MetS in the West (32.6%) and otherwise an overall low prevalence of MetS elsewhere (15.2–23.4%), but had a high prevalence of diabetes (8.7–12.7%). In three of the four regions, non-Hispanic black women had the highest prevalence of MetS among females of these three racial/ethnic groups (30.9–36.7%).

We next looked at regional differences in sex- and race/ethnicity-specific MetS severity *z*-scores, given known racial/ethnic discrepancies of ATP-III MetS in its relationship with insulin resistance and risk^12–16^. These MetS severity *z*-scores corresponded well in general with diabetes prevalence by region, with higher mean scores by sex and race/ethnicity in regions with high prevalence of diabetes (e.g., Hispanic males, with MetS scores 0.33–0.40—the highest of all groups—and diabetes prevalence of 9.19.1–11.1) and low mean scores in regions with low diabetes prevalence (e.g., among non-Hispanic white females). The exception was among non-Hispanic black males, who had overall low MetS severity *z*-scores (−0.20 to 0.08) in three of four regions with correspondingly high diabetes prevalence (9.3–10.3%).

Finally, Supplementary Table 2 provides prevalence of abnormalities in the individual MetS components by region. In assessing for specific MetS component abnormalities, we noted that the high MetS prevalence in the Midwest was associated with high prevalence of abnormalities in each individual component, with the Midwest having the highest prevalence of all regions for four out of five components (and second highest for BP elevations). These abnormalities in the Midwest were generally reflected among both males and females and among each racial/ethnic group. The South was in turn highest for BP elevations (particularly, among non-Hispanic blacks) and second highest for WC and low HDL (especially among Hispanics), while the West and Northeast were lowest for individual component abnormalities.

## Discussion

We found geographic variation in the prevalence of MetS, an important predictor of CVD, identifying areas where a large proportion of US adults are at risk. Overall, we found higher proportions of MetS-, obesity-, and diabetes-related risk in the center of the country, where 33–40% of individuals in the Midwest and South had MetS. This was in contrast to the New England, Mid-Atlantic, and Pacific divisions, where less than 30% of individuals had MetS. As opposed to prior reports which have suggested, for example, a stroke “belt” in the United States from the South up through the Ohio River Valley, these data and others begin to portray an obesity and MetS “zipper” extending from the South up through the Midwest. The reasons for these variations are unclear, though these differences appeared to parallel regional differences in the prevalence of abnormalities in the individual MetS components—with all five MetS component abnormalities being most common in the Midwest and South. Given that MetS is a stronger predictor of CHD than is obesity^10, 25^, these data may have implications for targeting surveillance and prevention to these more heavily affected areas, as suggested by the Institute of Medicine^3^. While more granular data are also important—providing glimpses into characteristics of neighborhoods at highest risk^26^—the current data nevertheless provide guidance for national public health policy makers regarding geographical areas of the country at very high metabolic risk and in need of additional attention.

While we are unaware of other such attempts to provide estimates of MetS-related risk by geographical area, our estimates of the prevalence of obesity by region are overall similar to those seen from other recent surveys. The behavioral risk factor surveillance system (BRFSS) relies on a telephone survey to collect height and weight measurements by self-report (with potential inaccuracy), reporting that in 2010 there were high concentrations of obesity prevalence in states in the South, where 8/8 states in the West South Central and East South Central had an obesity prevalence ≥30%; however, the BRFSS had overall lower estimates in the Midwest than we found, with only 2/12 states in the West North Central and East North Central having an obesity prevalence ≥30%^1^. A study based on measured height and weight estimates from the reasons for geographic and racial differences in stroke (REGARDS) data reported the highest obesity prevalence in the West North Central (41.3%), with prevalences in the range of 34–37% in the South and East North Central^1^, while a combination of BRFSS and REGARDS data reported the highest prevalence in the West North Central and West South Central^27^—very similar to what we found. Similarly, Slack et al. looked at multiple governmental sources for data on obesity prevalence at the county level and reported the highest concentrations in the South, with smaller pockets in states in the West North Central and East North Central^28^. Overall, these data support our findings of higher obesity prevalence in the South and Midwest.

With respect to estimates of CVD risk, Yang et al. evaluated a 10-year risk of CVD based on self-reported BRFSS data, finding that among males 3/4 states in the West South Central and 4/4 in the East South Central had a 10-year risk ≥15.1%, while risk was lower in the East North Central (4/5 states with 10-year risk ≥14.6%) and the West North Central (1/7 states with 10-year risk ≥14.6%)^29^. In another study, Yang et al. estimated CVD risk factors to determine “excess heart age,” finding the most concerning estimates in the South^30^. Death rates of coronary heart disease (CHD) by state published by the American Heart Association (AHA) identify similar geographic themes as we observed, with states of higher risk being located in the South and Midwest^2^. However, we noted the highest MetS prevalence in the West North Central division, where no state was in the highest category for CHD mortality^2^. This difference may be important in considering MetS as a marker of future CHD risk, identifying on-going needs for intervention in these states. Alternatively, this discrepancy between MetS and CHD mortality in this region may relate to higher healthcare access and earlier treatment^2^.

In considering intervention toward prevention of CVD, we noted potentially important differences by adult age group. Among young adults aged 20–39, there was a particularly high prevalence of obesity in the South (>35%) and of MetS in the South and West North Central (all >24%). While the West had overall more favorable prevalences of obesity and MetS compared to other regions, these prevalences among younger adults were not dramatically different from the Midwest, suggesting the potential for future increases in the prevalence of MetS and related disease risk as this younger group ages. In considering MetS as a risk indicator to prompt intervention, this higher prevalence of MetS among young adults in these regions may reflect the widespread nature of obesigenic lifestyle risks in this age group and the need to maintain lifestyle intervention efforts among young adults in all regions to prevent future CVD and diabetes.

The geographical variation in MetS did not on the surface appear to be due to differences in racial/ethnic demographics alone^16, 31^. While obesity is more prevalent among some racial/ethnic minorities^16, 32^, we found high prevalence of MetS in areas such as the West North Central division, which is >93.4% white, and low prevalence in the Mid-Atlantic and Pacific divisions, where 26.8% and 34% of individuals, respectively, are racial/ethnic minorities. Though we were limited by NCHS RDC restrictions that prevented reporting of racial/ethnic prevalence data in the nine US census divisions, we did note racial/ethnic differences between the four US census regions, with MetS particularly among non-Hispanic white women, who had a low prevalence of MetS in the West and Northeast (both <25%), but a high prevalence in the Midwest and South (both >32%). While the regional data were not in themselves designed to be nationally representative, these racial/ethnic differences could potentially prompt surveillance and diabetes prevention for specific at-risk groups.

There appeared to be a reasonable correspondence between diabetes prevalence and both ATP-III MetS prevalence and mean levels of a sex- and race/ethnicity-specific MetS severity *z*-score by subgroup and region. This MetS severity score was formulated in part because of noted discrepancies between ATP-III MetS and other risk factors for CVD and diabetes^12–16^. However, we noted that despite the high prevalence of diabetes among non-Hispanic black males by region, there were both lower-than-expected prevalence of ATP-III MetS and mean levels of MetS severity *z*-scores. These findings potentially reflect that non-Hispanic black males either continue to elude assessment of MetS-related risk or that they experience a high rate of non-MetS-related causes of diabetes, such as genetic relationships to beta-cell function^33, 34^. Our prior assessments of longitudinal risk related to MetS among non-Hispanic black males demonstrated similar long-term prediction of CVD as seen in whites^5^ and a stronger relationship between baseline MetS severity and prospective risk for diabetes^19^. Therefore, these relationships require further investigation.

Lower rates of obesity-related risk by geographical area in other surveys have been attributed to healthier lifestyle practices^2^. In assessing ideal cardiovascular health (a combination of physical activity, dietary, smoking, and health factors), the AHA reported that each of the states in New England and 9/13 West regional states were in the top two quintiles of ideal cardiovascular health practices compared to 0/20 states in the South or Midwest regions^2^. Therefore, policy changes in more highly affected regions, in addition to improving healthcare access, should consider targeting improved lifestyle practices across these populations to reduce future disease risk^3, 35^.

This study had multiple limitations, including the cross-sectional nature, which rendered us unable to conclude regarding causation between obesity, MetS, and diabetes. We were also limited by the relatively small numbers of participants in each US division, resulting in wide confidence intervals for many of the estimates reported and insufficient power to assess for changes in prevalence by division over time. Prior to 2007, sampling of Hispanic individuals who were not Mexican-Americans did not reflect the true proportion in the US population, meaning that prevalence estimates among Hispanic individuals may be excessively influenced by Mexican-American individuals. In addition, NHANES survey weights were designed to be nationally representative, not specifically representative of each US census region. Our intent in this analysis was to provide an ecologic study of the prevalence of obesity and MetS; further, analyses will be needed to determine how underlying contributors including diet, physical activity, smoking, socioeconomic factors, underlying inflammation, and healthcare access contribute to these differences by geographical area. This study had multiple strengths, being the first of its kind to assess geographic variation in MetS by US division and region and to explore related factors, including the individual MetS components and variation by age, sex, and race/ethnicity.

In conclusion, we noted geographic variation in MetS prevalence, with continued high need for preventive efforts in the center of the country among adults overall, and a fairly ubiquitous need for these efforts among young adults. Given the high rate of progression to CVD and diabetes among individuals with MetS, these data serve as a reminder for vigilance toward surveillance and lifestyle modification.

## Electronic supplementary material

Supplementary Figure 1

Supplementary Figure 2

Supplementary Table 2

Supplementary Table 1
