# Supplementary material for: Geographical variation in the prevalence of obesity, metabolic syndrome, and diabetes among US adults
Source: Nutr Diabetes. 2018 Mar 13;8:14. doi: 10.1038/s41387-018-0024-2 (PMC5856741; doi:10.1038/s41387-018-0024-2)
Supplement: Supplementary file 3 — Supplementary Table 2 [file 41387_2018_24_MOESM3_ESM.docx]

**Supplementary Table 2. Prevalences of Individual Metabolic Syndrome Component Abnormalities^†^ by Region & Demographics**

|  | **n** |  | **Blood Pressure Risk*** |  | **Elevated Fasting Glucose** |  | **Elevated Triglycerides** |  | **Low HDL Cholesterol** |  | **Elevated Waist Circumference** |
| --- | --- | --- | --- | --- | --- | --- | --- | --- | --- | --- | --- |
|  |  |  |  |  |  |  |  |  |  |  |  |
| **MIDWEST** (West North Central, East North Central) | | | | | | | | | | | |
| ***Overall*** | ***1943*** |  | ***34.7 (31.8, 37.6)*** |  | ***39.2 (35.7, 42.8)*** |  | ***30.2 (28.0, 32.5)*** |  | ***35.9 (31.9, 39.9)*** |  | ***54.9 (51.6, 58.2)*** |
| **Male** | **997** |  | **39.8 (35.7, 44.0)** |  | **48.8 (44.4, 53.1)** |  | **35.0 (30.9, 39.1)** |  | **33.8 (28.8, 38.8)** |  | **46.3 (42.4, 50.1)** |
| HISP |  |  | 29.0 (18.4, 39.5) |  | 48.7 (38.0, 59.4) |  | 44.2 (35.0, 53.5) |  | 32.3 (20.8, 43.8) |  | 28.5 (20.1, 36.9) |
| NHW |  |  | 40.3 (35.7, 44.9) |  | 49.8 (45.0, 54.7) |  | 36.0 (31.7, 40.4) |  | 34.6 (29.0, 40.3) |  | 48.4 (44.2, 52.6) |
| NHB |  |  | 40.5 (34.6, 46.5) |  | 37.7 (29.9, 45.4) |  | 19.7 (13.4, 26.0) |  | 26.2 (20.3, 32.1) |  | 33.3 (25.2, 41.5) |
| **Female** | **946** |  | **29.4 (26.2, 32.5)** |  | **29.4 (26.0, 32.7)** |  | **25.2 (21.6, 28.9)** |  | **38.1 (33.0, 43.2)** |  | **63.8 (59.9, 67.8)** |
| HISP |  |  | 20.9 (11.8, 30.0) |  | 20.2 (11.0, 29.4) |  | 29.1 (19.5, 38.7) |  | 45.4 (35.5, 55.3) |  | 66.0 (53.8, 78.3) |
| NHW |  |  | 28.8 (25.2, 32.4) |  | 29.4 (25.7, 33.1) |  | 25.9 (21.5, 30.4) |  | 37.8 (32.1, 43.4) |  | 62.9 (58.3, 67.6) |
| NHB |  |  | 38.5 (30.9, 46.1) |  | 33.0 (25.6, 40.4) |  | 17.1 (12.3, 21.9) |  | 38.2 (28.2, 48.3) |  | 72.0 (64.9, 79.0) |
| **NORTHEAST** (New England, Mid-Atlantic) | | | | | | | | | | | |
| ***Overall*** | ***1574*** |  | ***29.9 (26.8, 33.1)*** |  | ***38.4 (34.0, 42.7)*** |  | ***26.9 (24.1, 29.8)*** |  | ***30.6 (28.0, 33.1)*** |  | ***48.0 (44.1, 51.8)*** |
| **Male** | **789** |  | **33.2 (29.1, 37.4)** |  | **46.0 (40.0, 51.9)** |  | **33.4 (30.0, 36.8)** |  | **29.8 (26.3, 33.4)** |  | **40.40 (35.1, 45.7)** |
| HISP |  |  | 27.1 (19.2, 35.1) |  | 43.8 (35.3, 52.2) |  | 36.6 (28.2, 45.0) |  | 41.8 (33.4, 50.1) |  | 30.5 (23.4, 37.5) |
| NHW |  |  | 34.1 (29.4, 38.8) |  | 47.5 (40.8, 54.2) |  | 35.3 (31.5, 39.2) |  | 29.9 (26.0, 33.9) |  | 43.6 (38.2, 49.0) |
| NHB |  |  | 33.0 (25.8, 40.2) |  | 36.3 (29.3, 43.3) |  | 15.0 (10.6, 19.5) |  | 16.2 (8.3, 24.2) |  | 25.7 (20.6, 30.8) |
| **Female** | **785** |  | **26.5 (23.1, 29.8)** |  | **30.4 (26.9, 33.8)** |  | **20.1 (16.8, 23.4)** |  | **31.3 (27.5, 35.1)** |  | **55.9 (51.9, 60.0)** |
| HISP |  |  | 28.7 (18.9, 38.5) |  | 38.0 (30.4, 45.5) |  | 25.2 (16.7, 33.7) |  | 45.0 (34.8, 55.1) |  | 65.2 (54.7, 75.6) |
| NHW |  |  | 24.9 (20.6, 29.2) |  | 28.8 (24.6, 33.0) |  | 20.3 (16.2, 24.5) |  | 27.7 (23.1, 32.4) |  | 51.5 (45.7, 57.3) |
| NHB |  |  | 35.0 (27.4, 42.6) |  | 32.9 (28.7, 37.0) |  | 12.9 (7.2, 18.5) |  | 41.3 (36.0, 46.6) |  | 76.8 (71.7, 82.0) |
| **SOUTH** (West South Central, East South Central, South Atlantic) | | | | | | | | | | | |
| ***Overall*** | ***3820*** |  | ***35.1 (32.6, 37.7)*** |  | ***36.6 (33.8, 39.4)*** |  | ***27.9 (25.8, 30.0)*** |  | ***35.1 (33.0, 37.2)*** |  | ***51.1 (48.6, 53.6)*** |
| **Male** | **1893** |  | **39.0 (35.7, 42.3)** |  | **44.6 (41.2, 48.0)** |  | **32.0 (29.0, 35.0)** |  | **31.9 (29.6, 34.2)** |  | **41.6 (38.2, 44.9)** |
| HISP |  |  | 24.3 (19.6, 29.0) |  | 48.1 (42.4, 53.9) |  | 37.7 (33.4, 42.0) |  | 39.8 (34.4, 45.2) |  | 36.9 (30.8, 43.0) |
| NHW |  |  | 41.1 (36.5, 45.8) |  | 46.2 (42.1, 50.4) |  | 34.0 (30.0, 37.9) |  | 32.7 (29.2, 36.3) |  | 45.4 (40.9, 49.9) |
| NHB |  |  | 46.4 (41.0, 51.9) |  | 35.1 (30.0, 40.1) |  | 18.7 (15.1, 22.3) |  | 20.8 (17.0, 24.6) |  | 32.1 (27.3, 36.9) |
| **Female** | **1927** |  | **31.3 (28.6, 34.0)** |  | **28.8 (25.7, 31.9)** |  | **23.9 (21.5, 26.3)** |  | **38.1 (35.2, 41.0)** |  | **60.4 (56.9, 63.9)** |
| HISP |  |  | 19.2 (14.2, 24.2) |  | 32.7 (26.7, 38.8) |  | 25.2 (20.6, 29.8) |  | 47.5 (42.1, 52.8) |  | 65.5 (60.9, 70.0) |
| NHW |  |  | 30.4 (26.7, 34.1) |  | 26.8 (22.3, 31.4) |  | 27.9 (24.5, 31.2) |  | 38.1 (34.3, 41.9) |  | 55.4 (50.1, 60.8) |
| NHB |  |  | 42.5 (38.1, 47.0) |  | 31.9 (27.3, 36.6) |  | 10.9 (8.1, 13.7) |  | 31.7 (27.6, 35.8) |  | 72.3 (68.8, 75.9) |
| **WEST** (Pacific, Mountain) | | | | | | | | | | | |
| ***Overall*** | ***2489*** |  | ***28.0 (25.3, 30.7)*** |  | ***33.8 (31.0, 36.6)*** |  | ***29.5 (27.4, 31.6)*** |  | ***31.7 (28.9, 34.5)*** |  | ***48.2 (45.0, 51.4)*** |
| **Male** | **1265** |  | **31.1 (27.6, 34.6)** |  | **40.7 (36.4, 45.0)** |  | **36.1 (32.7, 39.5)** |  | **31.0 (27.6, 34.5)** |  | **36.9 (33.1, 40.8)** |
| HISP |  |  | 29.6 (26.0, 33.3) |  | 48.1 (43.8, 52.3) |  | 42.5 (37.5, 47.5) |  | 32.9 (29.0, 36.8) |  | 33.6 (29.1, 38.2) |
| NHW |  |  | 30.5 (25.9, 35.0) |  | 37.8 (31.6, 44.0) |  | 34.3 (30.2, 38.4) |  | 31.4 (26.5, 36.3) |  | 38.0 (32.4, 43.6) |
| NHB |  |  | 50.8 (39.7, 61.9) |  | 39.5 (30.5, 48.5) |  | 24.4 (12.9, 35.9) |  | 13.8 (6.0, 21.6) |  | 40.7 (31.7, 49.6) |
| **Female** | **1224** |  | **24.7 (21.9, 27.6)** |  | **26.6 (23.2, 30.0)** |  | **22.6 (20.1, 25.2)** |  | **32.4 (28.5, 36.3)** |  | **60.0 (55.7, 64.2)** |
| HISP |  |  | 18.1 (14.9, 21.4) |  | 31.2 (26.5, 35.9) |  | 23.6 (18.5, 28.8) |  | 42.7 (37.7, 47.8) |  | 67.9 (62.6, 73.1) |
| NHW |  |  | 25.8 (21.9, 29.7) |  | 24.7 (20.4, 29.0) |  | 23.1 (19.6, 26.5) |  | 28.4 (22.8, 34.1) |  | 56.1 (50.4, 61.7) |
| NHB |  |  | 41.9 (31.9, 52.0) |  | 30.8 (21.3, 40.3) |  | 12.0 (4.8, 19.3) |  | 37.1 (24.8, 49.4) |  | 74.7 (66.3, 83.2) |

^*^ Presented (for all MetS components): Weighted % and 95% CI

^†^ **Elevated Triglycerides:** Fasting Triglycerides ≥ 150

**Waist Circumference Risk:** Males, Waist Circumference ≥ 102 cm; Females, Waist Circumference ≥ 88 com

**Elevated Glucose**: Fasting Glucose ≥ 100

**Low HDL:** Males, HDL Cholesterol ≤ 40; Females, HDL Cholesterol ≤ 50

**Blood Pressure:** Systolic BP ≥ 130, or Mean Diastolic BP ≥ 85, or currently on antihypertensive medications
