# Supplementary material for: Geographical variation in the prevalence of obesity, metabolic syndrome, and diabetes among US adults
Source: Nutr Diabetes. 2018 Mar 13;8:14. doi: 10.1038/s41387-018-0024-2 (PMC5856741; doi:10.1038/s41387-018-0024-2)
Supplement: Supplementary file 4 — Supplementary Table 1 [file 41387_2018_24_MOESM4_ESM.docx]

**Supplementary Table 1: Obesity, MetS and Diabetes Prevalence and Mean Levels by Sex, Race/Ethnicity and US Region.**

|  |  |  |  | **Obesity** | |  | **ATP-III MetS** | |  | **Diabetes** | |  | **MetS Z-Score** | |  | **BMI** | |
| --- | --- | --- | --- | --- | --- | --- | --- | --- | --- | --- | --- | --- | --- | --- | --- | --- | --- |
|  |  | **n** |  | **%** | **95 CI** |  | **%** | **95 CI** |  | **%** | **95 CI** |  | **Mean** | **95 CI** |  | **Mean** | **95 CI** |
| **MIDWEST** (West North Central, East North Central) | | | | | | | | | | | |  |  |  |  |  |  |
| ***Overall*** |  | ***1943*** |  | ***35.5*** | ***(32.3, 38.6)*** |  | ***35.5*** | ***(32.9, 38.1)*** |  | ***8.9*** | ***(7.6, 10.3)*** |  | ***0.23*** | ***(0.17, 0.30)*** |  | ***28.92*** | ***(28.46, 29.38)*** |
| **Male** |  | **997** |  | **34.2** | **(30.4, 38.1)** |  | **37.9** | **(34.2, 41.7)** |  | **10.3** | **(8.4, 12.1)** |  | **0.34** | **(0.27, 0.41)** |  | **28.97** | **(28.55, 29.38)** |
| HISP |  | 126 |  | 30.0 | (20.5, 39.5) |  | 32.5 | (23.4, 41.6) |  | 11.5 | (4.3, 18.7) |  | 0.34 | (0.09, 0.59) |  | 27.63 | (26.67, 28.58) |
| NHW |  | 684 |  | 34.2 | (30.0, 38.4) |  | 39.6 | (35.3, 43.9) |  | 10.3 | (8.3, 12.3) |  | 0.37 | (0.09, 0.59) |  | 29.08 | (28.58, 29.59) |
| NHB |  | 187 |  | 36.5 | (28.2, 44.8) |  | 23.4 | (16.2, 30.6) |  | 9.4 | (5.7, 13.0) |  | 0.08 | (-0.11, 0.27) |  | 28.42 | (27.43, 29.41) |
| **Female** |  | **946** |  | **36.7** | **(32.9, 40.6)** |  | **32.9** | **(29.3, 36.5)** |  | **7.6** | **(5.6, 9.5)** |  | **0.13** | **(0.04, 0.21)** |  | **28.88** | **(28.24, 29.52)** |
| HISP |  | 105 |  | 39.4 | (29.4, 49.5) |  | 32.1 | (24.5, 39.7) |  | 10.7 | (5.3, 16.2) |  | 0.23 | (-0.04, 0.49) |  | 29.65 | (28.13, 31.17) |
| NHW |  | 656 |  | 35.2 | (30.7, 39.6) |  | 32.6 | (28.4, 36.9) |  | 7.0 | (4.8, 9.2) |  | 0.10 | (0.00, 0.21) |  | 28.59 | (27.87, 29.30) |
| NHB |  | 185 |  | 50.7 | (43.3, 58.1) |  | 36.0 | (28.0, 44.1) |  | 12.0 | (6.9, 17.0) |  | 0.29 | (0.07, 0.51) |  | 31.41 | (30.00, 32.82) |
| **NORTHEAST** (New England, Mid-Atlantic) | | | | | | | | | | | |  |  |  |  |  |  |
| ***Overall*** |  | ***1574*** |  | ***31.1*** | ***(27.7, 34.6)*** |  | ***29.6*** | ***(26.3, 32.9)*** |  | ***7.7*** | ***(5.8, 9.6)*** |  | ***0.07*** | ***(-0.00, 0.14)*** |  | ***28.05*** | ***(27.57, 28.52)*** |
| **Male** |  | **789** |  | **32.7** | **(28.7, 36.6)** |  | **33.1** | **(28.8, 37.3)** |  | **9.0** | **(6.3, 11.7)** |  | **0.24** | **(0.16, 0.33)** |  | **28.28** | **(27.73, 28.84)** |
| HISP |  | 154 |  | 31.7 | (24.2, 39.1) |  | 33.2 | (25.4, 41.1) |  | 11.1 | (4.8, 17.5) |  | 0.33 | (0.11, 0.55) |  | 28.35 | (27.58, 29.12) |
| NHW |  | 461 |  | 33.6 | (29.2, 38.0) |  | 35.3 | (31.2, 39.4) |  | 8.7 | (5.5, 11.8) |  | 0.29 | (0.19, 0.38) |  | 28.41 | (27.80, 29.02) |
| NHB |  | 174 |  | 26.3 | (22.1, 30.5) |  | 15.2 | (10.3, 20.1) |  | 9.3 | (5.8, 12.8) |  | -0.20 | (-0.31, -0.08) |  | 27.22 | (26.45, 28.00) |
| **Female** |  | **785** |  | **29.5** | **(25.3, 33.8)** |  | **26.0** | **(22.4, 29.7)** |  | **6.3** | **(4.5, 8.1)** |  | **-0.11** | **(-0.18, -0.05)** |  | **27.80** | **(27.26, 28.34)** |
| HISP |  | 193 |  | 32.8 | (25.1, 40.6) |  | 33.5 | (25.1, 41.8) |  | 15.7 | (6.8, 24.6) |  | 0.19 | (-0.08, 0.46) |  | 28.51 | (27.53, 29.49) |
| NHW |  | 414 |  | 25.8 | (20.0, 31.6) |  | 23.3 | (18.5, 28.2) |  | 4.1 | (2.1, 6.1) |  | -0.20 | (-0.30, -0.11) |  | 27.24 | (26.49, 27.99) |
| NHB |  | 178 |  | 52.3 | (46.6, 58.0) |  | 36.7 | (28.5, 44.9) |  | 11.3 | (6.3, 16.2) |  | 0.19 | (0.03, 0.35) |  | 31.02 | (30.19, 31.84) |
| **SOUTH** (West South Central, East South Central, South Atlantic) | | | | | | | | | | | |  |  |  |  |  |  |
| ***Overall*** |  | ***3820*** |  | ***35.3*** | ***(33.2, 37.5)*** |  | ***33.0*** | ***(30.6, 35.5)*** |  | ***7.4*** | ***(6.4, 8.4)*** |  | ***0.15*** | ***(0.10, 0.20)*** |  | ***28.72*** | ***(28.40, 29.05)*** |
| **Male** |  | **1893** |  | **32.7** | **(29.7, 35.7)** |  | **32.9** | **(30.1, 35.7)** |  | **8.1** | **(6.6, 9.5)** |  | **0.26** | **(0.21, 0.31)** |  | **28.52** | **(28.17, 28.87)** |
| HISP |  | 573 |  | 31.2 | (25.4, 37.0) |  | 31.9 | (28.4, 35.4) |  | 9.1 | (6.5, 11.8) |  | 0.37 | (0.29, 0.46) |  | 28.58 | (28.00, 29.16) |
| NHW |  | 737 |  | 32.9 | (29.2, 36.6) |  | 36.2 | (32.4, 40.0) |  | 7.0 | (5.0, 9.0) |  | 0.30 | (0.25, 0.36) |  | 28.51 | (28.09, 28.94) |
| NHB |  | 583 |  | 33.7 | (28.7, 38.7) |  | 21.7 | (17.1, 26.3) |  | 10.9 | (8.1, 13.8) |  | 0.02 | (-0.13, 0.16) |  | 28.49 | (27.82, 29.16) |
| **Female** |  | **1927** |  | **37.9** | **(35.0, 40.7)** |  | **33.1** | **(30.1, 36.2)** |  | **6.7** | **(5.3, 8.0)** |  | **0.04** | **(-0.03, 0.11)** |  | **28.92** | **(28.45, 29.39)** |
| HISP |  | 535 |  | 39.5 | (33.2, 45.7) |  | 34.4 | (28.8, 40.0) |  | 8.6 | (5.4, 11.8) |  | 0.14 | (0.00, 0.27) |  | 29.30 | (28.63, 29.96) |
| NHW |  | 737 |  | 32.2 | (27.8, 36.5) |  | 33.2 | (28.6, 37.8) |  | 4.4 | (2.7, 6.2) |  | -0.04 | (-0.15, 0.06) |  | 27.87 | (27.19, 28.54) |
| NHB |  | 655 |  | 54.4 | (50.9, 57.8) |  | 31.9 | (28.1, 35.7) |  | 12.3 | (9.8, 14.9) |  | 0.22 | (0.10, 0.33) |  | 31.89 | (31.31, 32.47) |
| **WEST** (Pacific, Mountain) | | | | | | | | | | | |  |  |  |  |  |  |
| ***Overall*** |  | ***2489*** |  | ***30.7*** | ***(28.2, 33.2)*** |  | ***29.6*** | ***(27.5, 31.7)*** |  | ***7.0*** | ***(5.3, 8.7)*** |  | ***0.10*** | ***(0.05, 0.16)*** |  | ***28.02*** | ***(27.63, 28.41)*** |
| **Male** |  | **1265** |  | **27.5** | **(24.7, 30.3)** |  | **32.5** | **(29.4, 35.7)** |  | **7.6** | **(5.5, 9.8)** |  | **0.24** | **(0.17, 0.31)** |  | **27.87** | **(27.47, 28.26)** |
| HISP |  | 675 |  | 30.3 | (26.3, 34.4) |  | 33.9 | (29.4, 38.5) |  | 10.8 | (7.7, 13.9) |  | 0.40 | (0.28, 0.52) |  | 28.38 | (27.81, 28.95) |
| NHW |  | 504 |  | 25.7 | (22.0, 29.4) |  | 32.0 | (28.0, 36.0) |  | 6.0 | (3.1, 8.9) |  | 0.19 | (0.10, 0.27) |  | 27.62 | (27.10, 28.13) |
| NHB |  | 86 |  | 37.4 | (27.3, 47.6) |  | 32.6 | (24.1, 41.1) |  | 12.7 | (6.3, 19.0) |  | 0.16 | (-0.14, 0.47) |  | 28.66 | (27.40, 29.92) |
| **Female** |  | **1224** |  | **34.1** | **(30.1, 38.0)** |  | **26.4** | **(23.6, 29.3)** |  | **6.4** | **(4.1, 8.7)** |  | **-0.04** | **(-0.12, 0.04)** |  | **28.18** | **(27.63, 28.73)** |
| HISP |  | 661 |  | 40.8 | (36.3, 45.4) |  | 29.8 | (25.6, 34.0) |  | 10.3 | (6.7, 13.8) |  | 0.19 | (0.05, 0.34) |  | 29.33 | (28.76, 29.89) |
| NHW |  | 467 |  | 30.3 | (25.3, 35.4) |  | 24.9 | (21.3, 28.6) |  | 4.8 | (2.2, 7.3) |  | -0.14 | (-0.23, -0.05) |  | 27.49 | (26.80, 28.18) |
| NHB |  | 96 |  | 52.4 | (40.5, 64.3) |  | 30.9 | (21.2, 40.5) |  | 10.0 | (3.3, 16.7) |  | 0.18 | (-0.08, 0.44) |  | 31.96 | (29.81, 34.11) |
